# Medication Adherence Apps: Review and Content Analysis

**DOI:** 10.2196/mhealth.6432

**Published:** 2018-03-16

**Authors:** Imran Ahmed, Niall Safir Ahmad, Shahnaz Ali, Shair Ali, Anju George, Hiba Saleem Danish, Encarl Uppal, James Soo, Mohammad H Mobasheri, Dominic King, Benita Cox, Ara Darzi

**Affiliations:** ^1^ Undergraduate Department of Medicine Imperial College London London United Kingdom; ^2^ Brighton and Sussex Medical School Brighton United Kingdom; ^3^ Division of Surgery Department of Surgery and Cancer Imperial College London London United Kingdom; ^4^ Institute of Global Health Innovation Imperial College London London United Kingdom; ^5^ Imperial College London South Kensington Campus London United Kingdom

**Keywords:** medication adherence, patient compliance, mobile apps, telemedicine, smartphone, reminder systems, treatment outcome

## Abstract

**Background:**

Medication adherence is an expensive and damaging problem for patients and health care providers. Patients adhere to only 50% of drugs prescribed for chronic diseases in developed nations. Digital health has paved the way for innovative smartphone solutions to tackle this challenge. However, despite numerous apps available claiming to improve adherence, a thorough review of adherence apps has not been carried out to date.

**Objective:**

The aims of this study were to (1) review medication adherence apps available in app repositories in terms of their evidence base, medical professional involvement in development, and strategies used to facilitate behavior change and improve adherence and (2) provide a system of classification for these apps.

**Methods:**

In April 2015, relevant medication adherence apps were identified by searching the Apple App Store and the Google Play Store using a combination of relevant search terms. Data extracted included app store source, app price, documentation of health care professional (HCP) involvement during app development, and evidence base for each respective app. Free apps were downloaded to explore the strategies used to promote medication adherence. Testing involved a standardized medication regimen of three reminders over a 4-hour period. Nonadherence features designed to enhance user experience were also documented.

**Results:**

The app repository search identified a total of 5881 apps. Of these, 805 fulfilled the inclusion criteria initially and were tested. Furthermore, 681 apps were further analyzed for data extraction. Of these, 420 apps were free for testing, 58 were inaccessible and 203 required payment. Of the 420 free apps, 57 apps were developed with HCP involvement and an evidence base was identified in only 4 apps. Of the paid apps, 9 apps had HCP involvement, 1 app had a documented evidence base, and 1 app had both. In addition, 18 inaccessible apps were produced with HCP involvement, whereas 2 apps had a documented evidence base. The 420 free apps were further analyzed to identify strategies used to improve medication adherence. This identified three broad categories of adherence strategies, *reminder*, *behavioral*, and *educational*. A total of 250 apps utilized a single method, 149 apps used two methods, and only 22 apps utilized all three methods.

**Conclusions:**

To our knowledge, this is the first study to systematically review all available medication adherence apps on the two largest app repositories. The results demonstrate a concerning lack of HCP involvement in app development and evidence base of effectiveness. More collaboration is required between relevant stakeholders to ensure development of high quality and relevant adherence apps with well-powered and robust clinical trials investigating the effectiveness of these interventions. A sound evidence base will encourage the adoption of effective adherence apps, and thus improve patient welfare in the process.

## Introduction

### Adherence Problems and Opportunities

In the age of advanced medical treatments, a significant obstacle to improve outcomes is the failure of patients to adhere to medication prescribed by their physicians. Medication adherence and compliance can be defined as the “act of (the patient) conforming to the recommendations made by the provider with respect to timing, dosage, and frequency of medication taking” [1].

A World Health Organization report on adherence to long-term therapies suggests that patients adhere to only 50% of drugs prescribed for chronic diseases in developed nations, a figure that is even lower in developing countries. The same report also highlights two major consequences of nonadherence: (1) suboptimal health outcomes for patients and (2) rising health care costs [2].

The rapid growth of mobile technologies and their uptake by consumers worldwide presents opportunities and solutions that attempt to address the problems within health care systems. This use of portable technology in health care is called mobile health (mHealth) [3]. With an estimated 2 billion smartphone users worldwide [4] and apps becoming a ubiquitous part of people’s lives, it is no surprise that there are over 97,000 mHealth apps available on various app repositories, and the mHealth app market is projected to reach a revenue of US $26 billion by 2017 [5]. The fifth biggest category of mHealth apps relate to medical condition management [5]. This category contains apps, which help users adhere to medication and monitor intake [5].

Previous studies on adherence apps have focused on the prevalence of behavior change techniques, ideal features, health literacy, content, and usability [6-9]. A literature review found only 14 papers and 4 app-related reports in which the “majority of reviewed studies showed a positive impact on the use of existing mobile apps for medication adherence” [10]. A review of diabetic self-management apps showed that there is a gulf between diabetes self-management guidelines and the features available on apps to meet these guidelines [11]. However, no thorough review has been conducted to evaluate all adherence apps with respect to their degree of evidence base, or medical professional involvement in their development.

### The Objective

The aim of this study was to review the currently available medication adherence apps in the two largest app repositories, the Apple App Store and the Google Play Store, in terms of their evidence base, medical professional involvement in development, and strategies used to facilitate behavior change and improve adherence.

## Methods

### Initial Search

Relevant medication adherence apps were identified by interrogating the Apple App Store and Google Play Store using the primary search terms, which are “medication,” “medicine,” “pill,” “drug,” and “tablet,” combined with secondary search terms, which are “reminder,” “alarm,” “manager,” “tracker,” “list,” “organizer,” “helper,” “compliance,” “adherence,” and “accordance.” The search and review took place in April 2015.

Any identified app designed to facilitate patient adherence to medications was included. The term *medication* in this study was defined as physical pharmacological treatment only *.* Apps designed primarily for nonpatient groups, for example, health care professionals (HCPs), and those providing no adherence support were excluded. Apps that provided lists of medicines or conditions such as encyclopedias were excluded. Apps that were available as a larger bundle (groups of up to 10 apps sold together at a reduced price) were also excluded. These apps were all tested individually, hence not requiring download of the bundle. Apps in languages other than English were excluded.

Data were extracted for each app from the app repository overview and the developer’s website. Not all apps provided a website address; therefore, for a number of apps, information was gleaned from testing alone. Relevant data items included (1) documentation during the development of the app, and (2) availability of evidence base pertaining to the app (either relating to its design and development, or its efficacy). Other datasets were collected but found irrelevant to analysis; these are stated in Multimedia Appendix 1.

HCP involvement was defined as any individual working within the health care industry who was directly involved with the distribution or prescription of medication to patients. Hence, this included physicians, pharmacists, and nurses.

Evidence base was defined as an app providing data on trials or studies that are carried out utilizing the app to indicate effectiveness. This was only accepted once a report, study, or trial was seen by testers to validate the claim.

### Testing Phase

Free apps were downloaded for further testing to explore the specific adherence strategies utilized by apps to promote medication adherence (eg, alarms and push notification reminders). Any additional feature not contributing specifically to adherence but designed to enhance user experience was also documented (eg, pharmacy locator function and refill reminder). In the case of inaccessible and paid apps, the identification of features was based on the app description and publisher website. Inaccesible apps were those that could only be accessed with authorization provided by a specific health care organization, pharmacy or health care provider.

Four researchers performed the data extraction. They identified the adherence methods used by apps and within those features, which subsets were utilized. Once a feature was identified, it was placed within an Excel spreadsheet alongside the app’s name, which all reviewers had access to.

To provide reliability throughout testing, definitions for each adherence feature were established and agreed upon by all 4 reviewers.

A devised medication regime was input into all identified apps, and this was used by all 4 reviewers to test the apps in terms of adherence mechanisms utilized. If there was any uncertainty or doubt about an app’s adherence mechanisms, it was resolved by consensus among the 4 reviewers.

All 4 reviewers tested the first 10 apps identified within the Apple App Store and the Google Play Store independently. Results of individual reviewers were then compared, and the interrater reliability was determined using the Fleiss Kappa coefficient.

The remaining apps were then equally allocated among reviewers. Data were extracted and placed into a spreadsheet for analysis.

During testing, any app that did not function was excluded, and details were kept in a separate spreadsheet, including the reasons for nonfunctioning. Only apps that functioned and fulfilled an adherence function were included for testing.

## Results

### Interrater Reliability

Interrater reliability between the 4 testers was calculated using the Fleiss Kappa coefficient (reproducibility between more than 2 testers). A sample of 20 apps (10 from each respective app store) was used, which resulted in a coefficient of .61 (SE 0.078; 95% CI 0.46-0.76). This suggests good reproducibility between the reviewers according to the Landis and Koch rules for interpreting Fleiss Kappa coefficient values [12].

### App Identification

**Figure 1:**
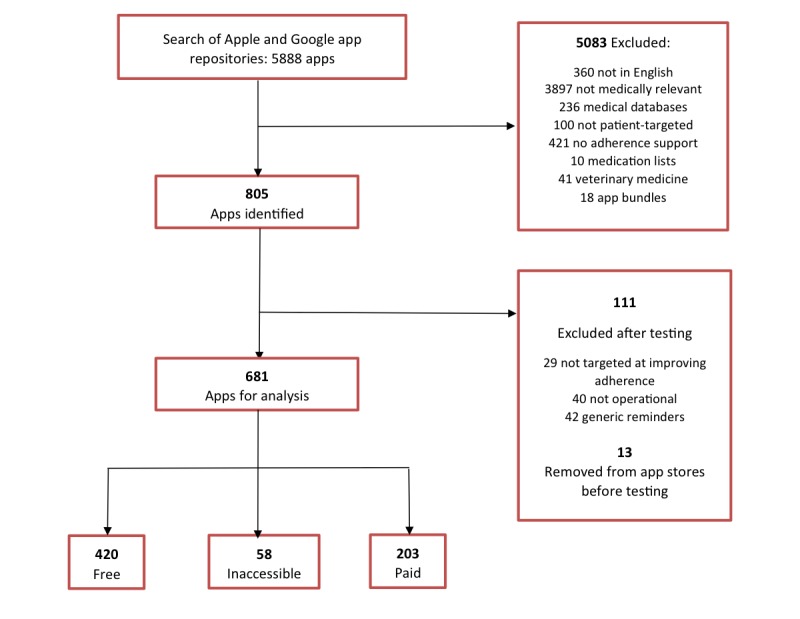
Flowchart of identification of applications.

The app repository search identified 5888 apps, of which 5207 apps were excluded, leaving 681 apps for analysis (see Figure 1).

The majority of those excluded were medically not relevant; these included various apps, for example, video games, magazine apps, to-do list, and wall paper apps.

Where possible data were extracted through app testing and from developer websites, where apps had a linked website. Of the free apps, 260 apps provided a website, with 160 apps providing no website.

Moreover, 186 apps were solely found in the Google Play Store, 136 apps originated from the Apple App Store, and 98 apps were found in both repositories.

Download Stats were only available for Google Play Store apps. Of the 284 apps available for analysis, 168 (59.2%) had fewer than 10,000 downloads (<10,000), 63 (22.2%) apps had over 10,000 downloads (>10,000), and 53 (18.7%) apps had no available Download Stat.

### Health Care Professional Involvement in App Development and Evidence Base

Of the 420 free apps, 13.6% (57/420) of the apps were developed with involvement from HCPs in the medical or pharmaceutical industry.

Meanwhile, mention of an evidence base (either in relation to the development process or of app effectiveness) was identified in only 1.0% (4/420) of apps. One app referenced trialing and testing by a patient panel from myhealthapps.net (network). Another app described following evidence-based patient safety practices recommended by the Minnesota Alliance for Patient Safety. The final 2 of the 4 apps specifically highlighted patient pilots and clinical trials in which their apps were used and have published the data.

Of the paid apps, 4.4% (9/203) of apps had HCP involvement in development, 0.5% (1/203) of apps had a documented evidence base, and 0.5% (1/203) of apps had both. The single evidence-based app was subjected to a randomized controlled trial and proved to be beneficial with 95% of participants adhering to medication. There was also one app, which was supported by the National Health Service Health Apps Library.

In addition, 31% (18/58) of inaccessible apps were produced with HCP involvement, whereas 3% (2/58) of apps had a documented evidence base. One of the 2 apps had produced a case study based on their app; however, this was not available for access. The other had developed a case study with a partnered company using their work, detailing the benefits of the companies offering. There were no clinical trials.

### Download and Testing Phase

A total of 420 free apps were downloaded and further analyzed to identify strategies used to improve medication adherence. This led to the identification of three broad categories of adherence strategies: *reminder*, *educational*, and *behavioral*. The reminder category was defined as any strategy that acted to inform the user that it was time to take medication. The educational category was defined as any strategy that better informs patients regarding the importance of medication adherence. The behavioral category was defined as behavior change strategies used by apps to encourage adherence. A total of 59.5% (250/420) of apps utilized a single method, 35.5% (149/420) of apps used two methods, and only 5.2% (22/420) of apps utilized all three methods to improve adherence. The breakdown of apps according to the methods used is shown in Table 1.

**Figure 2:**
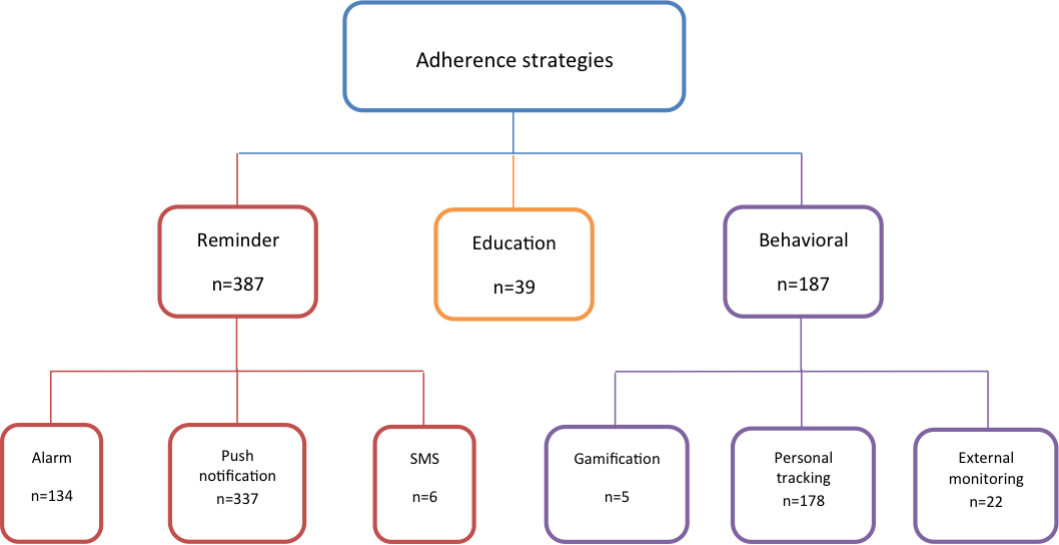
Taxonomy of identified adherence strategies.

It was apparent following the download and testing of apps that the behavioral and reminder categories could be further subdivided in line with the various identified techniques used by apps. This allowed the development of a taxonomy of adherence strategies utilized by apps (Figure 2).

The reminder classification was subdivided into three subcategories: (1) *Alarm*, which referred to the mobile device providing an audio alert at a preset time *,* (2) *Push Notification*, which was an internal message appearing on the mobile device at a set time indicating need to take medication, and (3) *Short Messaging Service (SMS)*, which delivered a text message indicating a reminder for taking medication at a set time.

The subcategories for the behavioral classification were (1) *External Monitoring*, (2) *Personal Tracking*, *and* (3) *Gamification.* External monitoring was a strategy that enabled users to send adherence-related data to third parties (such as family, friends, or HCP). Personal tracking referred to any capacity of the app to allow users to track their medication taking and create a record of it. Gamification was defined as any method to provide video game-like elements to the medication-taking process to encourage good medication adherence. An example applied to medication adherence would include in app rewards for high levels of adherence, such as badges or providing a level scheme.

#### Reminder

Almost all apps utilized a reminder function of some sort to facilitate adherence to medications; the number totaled 387 apps, amounting to 92.1% (387/420) of all apps tested. The largest subcategory was *Push Notifications*; 80.2% (337/420) of apps utilized this method. *Alarms* were ranked second with 134 apps, and finally very few, 1.4%, (6/420) of apps incorporated *SMS Reminders*. A breakdown of the app numbers utilizing various reminder subcategories are provided in Table 2.

**Figure 3:**
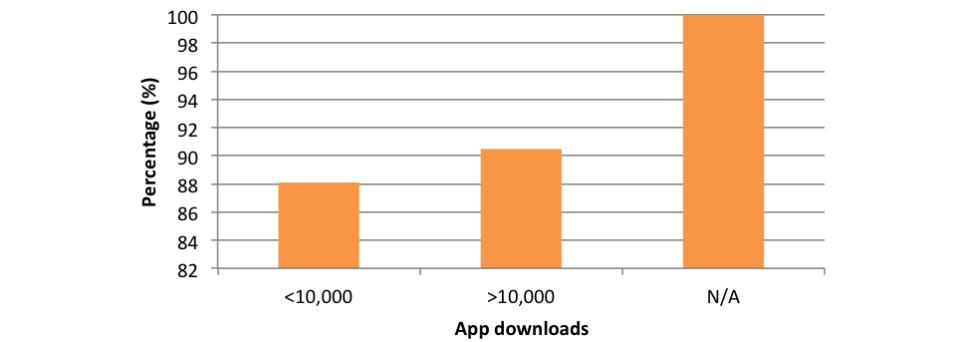
Chart comparing reminder function percentage according to downloads.

Reviewing the reminder function according to the number of downloads revealed in the <10,000 downloads group that 88.1% (148/168) of apps utilized a reminder function. In the over >10,000 downloads group, 90% (57/63) of apps possessed a reminder function, and in the group where download data were unavailable, 100% (53/53) of apps utilized a reminder function (Figure 3). These results relate only to apps within the Google Play Store.

**Figure 4:**
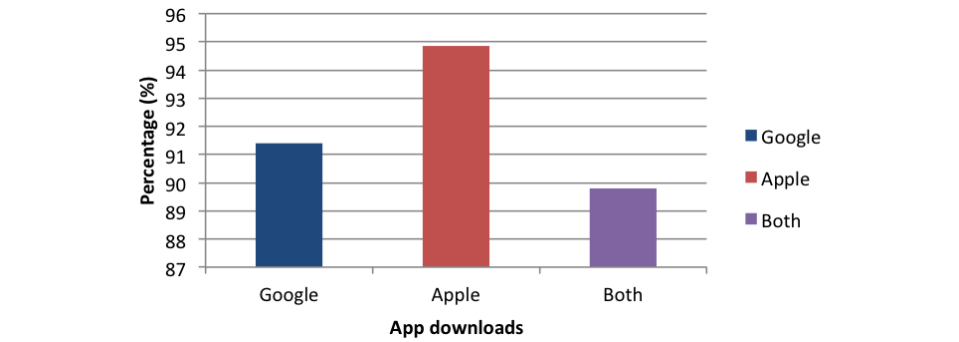
Chart comparing reminder function percentage among apps in different app stores.

Comparison of apps according to app repository revealed that 170 (91.4%) apps of 186 Google Play Store only apps, 129 (94.9%) apps of 136 Apple App Store only apps, and 88 (89.8%) apps of 98 apps in both store utilized a reminder function (Figure 4).

**Table 1:** Numbers of apps adopting the various adherence strategies.

Strategy	Number of apps
Reminder	220
Behavioral	28
Education	1
Reminder, behavioral	133
Reminder, education	12
Behavioral, education	4
Reminder, behavioral, education	22
Total	420

**Table 2:** Number of apps adopting reminder strategies.

Strategy	Number of apps
Alarm	48
Push notifcation	248
Short messaging service	2
Alarm, push notification	85
Alarm, short messaging service	0
Short messaging service, push notification	3
Alarm, short messaging service, push notification	1
Total	387

#### Behavioral

This category was the second largest, with 44.5% of apps (187/420) utilizing one or more of the three behavioral technique subcategories. A total of 42.4% of apps (178/420) used the *Personal Tracking* feature. In addition, 95.1% (174/178) of apps using a behavioral strategy incorporated personal tracking.

Comparatively, 22 apps (5.2%) used a form of *External Monitoring*. Last were apps using *Gamification.* Analysis showed that 5 apps (1.2%) utilized this strategy. A breakdown of the app numbers utilizing various behavioral subcategories is provided in Table 3.

**Figure 5:**
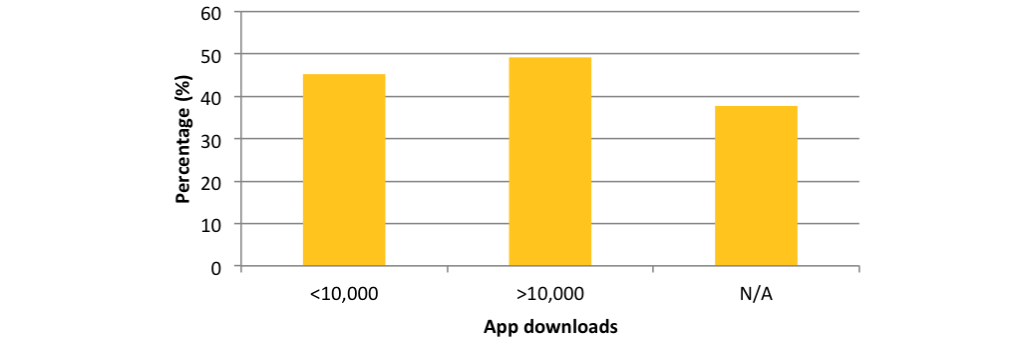
Chart comparing behavioral function percentage according to downloads.

Comparing by number of downloads (Google Play Store available apps): in the <10,000 group, 45.2% (76/168) of apps; in >10,000 group, 49% of apps (31/63); and in apps where download data were not available, 37% of apps (20/52) utilized a behavioral function (Figure 5).

**Figure 6:**
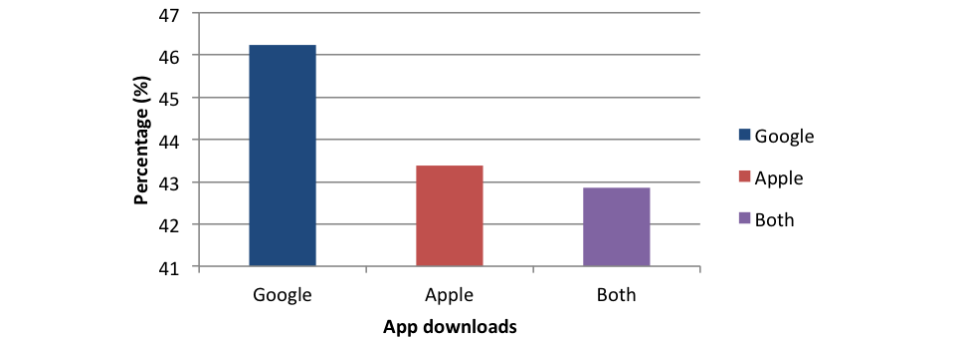
Chart comparing behavioral method percentage among apps in different app stores.

Comparison of apps according to app store revealed that 46.2% (86/186) of Google Play Store only apps, 43.4% (59/136) of Apple only apps, and 43% (42/98) of apps in both stores utilized a behavioral function (Figure 6).

#### Education

**Figure 7:**
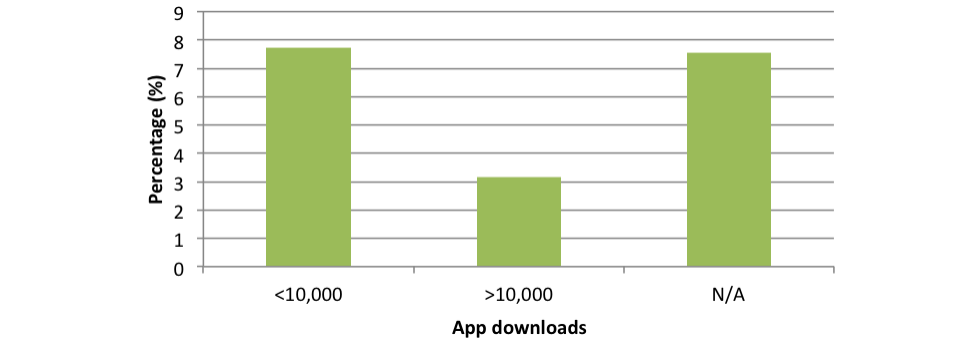
Chart comparing education method percentage according to downloads.

A total of 39 apps used education as a method. Comparing by number of downloads (Google Play Store available apps): in the <10,000 group, 7.7% of apps (13/168); in >10,000 group, 3% of apps (2/63), and in apps where download data were not available, 8% of apps (4/53) utilized education as a method (Figure 7).

**Figure 8:**
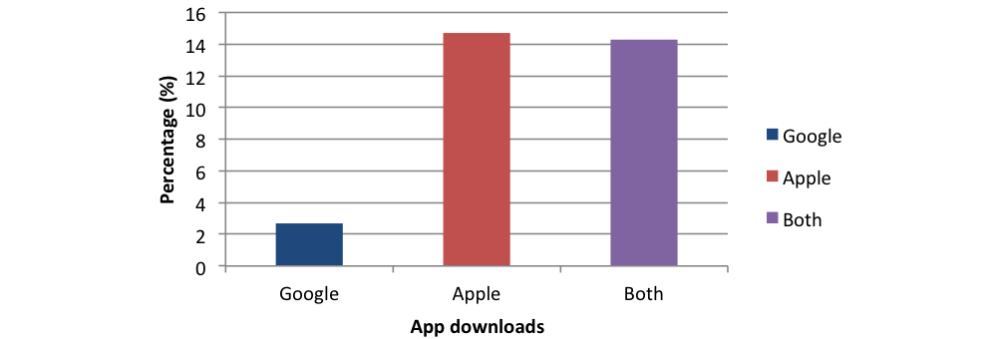
Chart comparing educational method percentage among apps in different app stores.

Comparison of apps according to app repository revealed that 2.7% of (5/186) Google Play Store only apps, 14.7% (20/136) of 136 Apple only apps, and 14% (14/98) of apps in both stores utilized education as a method (Figure 8).

### User Features

Through testing, various additional user features were identified; these are listed in Table 4.

**Figure 9:**
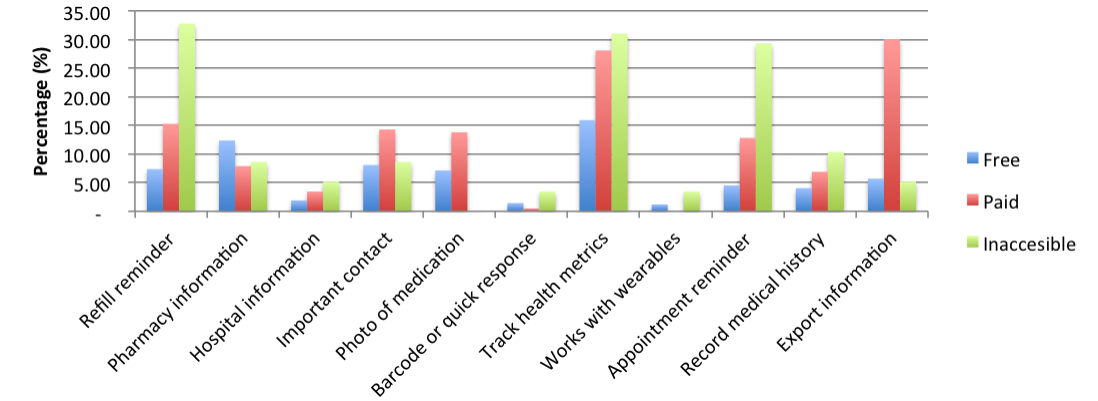
Chart comparing user features across payment modalities.

Figure 9 provides a breakdown of the offerings of these additional user features according to whether apps were free, inaccessible, or paid. A large number (224/681) of apps did not offer any user features: 38.3% (161/420) of the free apps, 27.6% (56/203) of the paid apps, and 12% (7/58) of the inaccessible apps.

**Table 3:** Number of apps adopting behavioral strategies.

Strategy	Number of apps
Gamification	1
Personal tracking	161
External tracking	8
Gamification, personal tracking	3
Gamification, external tracking	0
Personal tracking, external tracking	13
Gamification, personal tracking, external tracking	1
Total	187

**Table 4:** User features offered by apps.

User feature	Description of feature	Number of free apps with user feature
Track other health metrics	Such as blood pressure	67
Pharmacy information	Information relating to nearby pharmacies, such as contact information or location	52
Important contacts	Can input information relating to pharmacist, doctor, or emergency contact in the app	34
Refill reminder	An alarm or reminder relating to when the user requires refilling of their medication	31
Photo of medication	Add a picture of the medication or select image from existing gallery to place next to medication on app	30
Export information from app	Can email or send information on medication or adherence record to another person, such as a health care provider	24
Appointment reminder	Reminds you of medical appointments	19
Record medical history	Can act as an electronic medical record by inputting medical history	17
Hospital information	Information relating to nearest hospital, contact information, and location	8
Barcode scanner	Scans barcode and automatically inputs medication according to the barcode	6
Work with wearables	Compatibility with wearable technology	5

## Discussion

### Principal Findings

To our knowledge, this is the first study to systematically and exhaustively review all currently available medication adherence apps on the two largest app repositories. Dayer et al [13] is the only comparable study of this nature to look at a wide number of medication adherence apps and explore desirable features. However, only 10 of the highest rated apps were downloaded and user tested compared with 420 apps in this review. This possibly reflects the rapid expansion in mHealth app release year on year [5].

One of the most important findings of this study is the concerning lack of HCP involvement in app development (84/681, 12.3%) and the limited evidence base related to the development and use of such apps (8/681, 1.2%). App reviews focusing on other medical fields have reported similar findings such as colorectal conditions [14], vascular conditions [15], urology [16], orthopedic sports medicine [17], hernias [18], obesity [19], ophthalmology [20], and pain management [21]. Although the involvement of HCPs in app development does not necessarily guarantee app efficacy, it is likely to provide greater insight into patient needs and is suggestive of more reliable content and higher quality.

Of the 8 identified evidence-based apps, only 3 apps related specifically to clinical trials investigating app efficacy (in terms of an improvement in medication adherence rates). In the current era of evidence-based practice, robust evidence supporting the use of app-based interventions is necessary if there is to be widespread HCP buy-in to apps or if apps are to be prescribed and reimbursed by health care systems in the future, in much the same way as drugs currently are. The limited prevalence of evidence-based apps may, in part, be explained by the inherent tension that exists between the slow-paced and arduous nature of gold-standard health care intervention evaluation methodologies (such as the randomized controlled trial) and the fast-paced and evolving nature of app technologies [22,23]. Newer, faster evaluation methodologies may be required to address such challenges going forward.

The testing of adherence apps undertaken in this study has enabled us to create a taxonomy of strategies that have been utilized by such apps to promote behavior change and adherence. The wider adherence literature describes two broad types of nonadherence among patients [24]: (1) unintentional—where patients intend to take their prescribed medicines but ultimately do not (eg, due to forgetfulness) and (2) intentional—where patients make an active decision not to take their medicines. The results of this study indicate that the majority of currently available adherence apps utilize strategies targeting unintentional nonadherence, such as reminders. Push notifications in particular were the predominant technique utilized. Interestingly, only 1.4% (6/420) of apps reviewed in this study used SMS as a means of sending reminders, despite existent evidence demonstrating the effectiveness of SMS reminders in improving adherence [25]. One review concluded that as reminder apps serve a very similar function to but have a broader range of functionality than SMS messaging; the potential for such apps to improve medication adherence will be at least equal to, if not greater than, SMS reminders [13]. This provides a potential explanation for the demonstrated lack of SMS utilization compared with other reminder methods.

Educational strategies, which may be of potential benefit in both unintentional and intentional nonadherers, were also underutilized, despite evidence demonstrating that increasing patient knowledge regarding medicines and the importance of taking prescribed medicines improves adherence [26].

External monitoring was another poorly utilized adherence strategy. This strategy allows third parties to receive adherence information of the patient, giving them greater opportunity to become more actively involved and integrated with patient care. This may be of particular benefit in those with chronic conditions. Although the overall utilization of external monitoring was low, prevalence in the inaccessible groups of apps was much higher (28% [16/58] vs 5.2% [22/420]), highlighting how certain clinics and pharmacies are taking on the responsibility of monitoring and promoting adherence of their patient populations through the use of apps.

Gamification was the least commonly utilized adherence strategy, with just 1.2% (5/420) of apps utilizing this technique. It is an umbrella term used to describe “the use of video game elements in nongaming systems to improve user experience and user engagement” [27]. The evidence base in support of gamification as a method of promoting behavior change is growing. One systematic review demonstrated that 69% of psychological therapy outcomes and 59% of physical therapy outcomes were improved by video games; results did not differ across age groups [28]. The target markets for the gamification apps identified in this study were not age specific; tailoring apps to an age demographic may allow for the more effective use of gamification. *Pain Squad* is an example of an effective gamification app targeted at a younger audience; it is used to document pain levels in children with cancer and had high compliance and satisfaction ratings [29]. The positive uptake among children and adolescents may be replicable for medication adherence.

Aside from the various adherence strategies provided by apps, a large proportion also offered a host of additional user features and functionality, falling into one of 11 categories. The most common features were health metric tracking, medication refill reminders, pharmacy information, and directories of health care service contacts. The least prevalent features were barcode scanning, connecting with wearable technologies, and hospital information provision. In general, user features were found to be more prevalent among paid apps, offering a more comprehensive service for the individual downloading the app and justifying the cost price.

Although few identified apps provided barcode scanning (using digital quick response code technology to capture the relevant identifier on a drug packet), such technology has been demonstrated to reduce medical error rates, thereby promoting patient safety [30]. Consequently, the provision of barcode scanning within adherence apps should be encouraged.

Finally, the literature highlights that nonadherence is particularly common among the elderly, who are often on multiple, life-long medicines [31,32] and may suffer with memory impairment [32,33]. It stands to reason, therefore, that this demographic potentially stands to gain the most from app-based adherence interventions. Unfortunately, however, this same demographic is less familiar and interested in such technologies and also more likely to suffer from physical ailments such as limited dexterity [34,35]. However, more recent evidence suggests that this trend is changing as interest increases in mHealth [36]. Consequently, it is imperative that developers offer enhanced accessibility features to increase the reach of apps into the older age groups. In this regard, a number of reviewed apps offered the ability to increase the displayed font size and text fields and provided a larger keypad for data entry.

### Limitations

Several limitations were identified in this study. First, although we were able to download and test free apps to identify the adherence strategies that they utilized, we were unable to download and test paid apps because of lack of funding. From app repository descriptions, it appears that paid apps offered additional features and functionality and the ability to download such apps may have yielded further useful insights around the strategies used by apps to promote adherence. Similarly, we were also unable to download and test inaccessible apps, which required log-in credentials from an affiliated health care organization or clinic.

As a consequence of the dynamic nature of the mHealth apps market and the rapid turnover of apps, several apps initially identified for inclusion in this review were subsequently withdrawn from app repositories rendering potentially influential data gleaned from such apps redundant.

Finally, because of the rapid production and release of new apps, we acknowledge that as this review was performed, new adherence apps will have been released that have not been included in this study.

### Future Research

We have highlighted two main potential areas for future research. First, although we have used HCP involvement as a surrogate market for app quality, other markets are also likely to be important such as patient involvement in the creation of apps. Further research involving focus groups and qualitative assessment of apps with patients will help in addressing this issue.

Second, we have focused on all medication adherence apps irrespective of disease condition to get a broad overview of the market. Future research may therefore focus on apps designed for adherence in specific disease contexts.

### Conclusions

This app repository review demonstrates a concerning lack of HCP involvement in app development. Greater collaboration is required among app developers, HCPs, academics, behavioral scientists, and end users to ensure the development of high-quality, relevant adherence apps.

The results have also identified that the vast majority of current adherence app offerings on repositories lack any evidence base of effectiveness. In this regard, well-powered and robust clinical trials investigating the effectiveness of these interventions are needed going forward. Such evidence will enable HCPs to prescribe an adherence app whenever they are prescribing a medicine, thereby resulting in widespread adoption among patients.

## Appendix

Multimedia Appendix 1 Supplementary table.

## Abbreviations

HCP: health care professional
mHealth: mobile health
SMS: short messaging service
