# Supplementary material for: Medication Adherence Apps: Review and Content Analysis
Source: JMIR Mhealth Uhealth. 2018 Mar 16;6(3):e62. doi: 10.2196/mhealth.6432 (PMC5878368; doi:10.2196/mhealth.6432)
Supplement: Multimedia Appendix 1 [file mhealth_v6i3e62_app1.pdf]

**<u>Data term extracted</u>**
Application name
Developer
Year of release
Last update (if available)
Store available in
Cost
In app purchases
Application rating (if available)
Website (if available)
